# 

**Published:** 1914-11-07

**Authors:** 


### CONTENTS.

| | PAGE |
|---|---|
| **Medical Men as Prisoners of War ... ...** | 121 |
| **The Institutional Future ... ... ... ...** | 122 |
| **Hospital and Institutional News ... ... ...** | 123 |

Our Readers and the Belgian Wounded—The Price of Calais—The Wreck of the Hospital Ship *Rohilla*—A Surviving Doctor's Experiences—Alleged Red Cross "Plot" in Ireland—Free Choice of Panel Doctor—The Thick End of the Wedge—Education by Emergency—Repatriating a Mental Patient—War's Effect on Mental Hospital Expenses—The Care of the Belgian Refugees—A French Mental Hospital War Story—A House of Quiet—The Secretaryship of the General Hospital, Birmingham—The American Motor Ambulance Corps—The Vindication of a Medical Superintendent—Notification Fees in Pulmonary Tuberculosis—A Treasury Concession to Military Hospitals—Bradford Royal Infirmary and the Corporation Scheme—The North Staffordshire Infirmary Staff—Mental Hospital Staffs and War Losses—The Architecture of Emergency Hospitals—Dispensing Fees and "Sweating Prices"—A Remarkable Cottage Hospital Treasurer—The Art of Appeal in War Time—Turkey and the Drug Market—To Our Readers.

[*See over.*]

### CONTENTS—(*continued*).

PAGE

**The Work of the R.A.M.C. in the War** ... 129
The Regimental Medical Officer's Duties.

**Medical Appointments** ... ... ... ... 130

**The Naval Red Cross Train in London** ... 131
Wounded Transferred to *Dreadnought* Hospital: Excellence of the Navy Transport System.
Great Northern Central Hospital.

**Hospitals and the War** ... ... ... ... 132
What the London Hospitals are Doing.
**Work for the Wounded of the Allied Armies**
More Convoys at Cambridge—Huddersfield: Royal Infirmary—Norwich: An Urgent Summons—North Staffordshire Infirmary, Stoke-on-Trent.

**The Influx of Wounded at Leicester** ... ... 134
Work of the Heaviest Week of the War.

**Personalia** ... ... ... ... ... ... 137

**Dr. Ellwood on Medical Gardening** ... ... 137

PAGE

**Reports on Hospitals of the United Kingdom**— 135
By SIR HENRY BURDETT, K.C.B., K.C.V.O.
Faulty Administration from Ignorance of Modern Hospital Progress.

**Buildings Contemplated** ... ... ... ... 137

**Round the World** ... ... ... ... ... 138

Fellow-Workers and the Roll of Honour.

**The Institutional Library** ... ... ... ... 139
"Heretical" Lectures—A Medical Novel—A Companion for Ward Work—The Sanatorium as a School—A Guide to Medical Electricity.

**The Institutional Worker** ... ... (Suppl.) 1
Electrical Cooking for Hospitals.
Questions for November.
The Sick and in Need.
Miscellaneous.
War Matters.
Employment and Training.

